# Molecular Typing of *Pseudomonas aeruginosa* Isolates Collected in Abidjan Hospitals (Côte d’Ivoire) Using the Multiple-Locus Variable Number of Tandem Repeats Method

**DOI:** 10.3390/diagnostics14202284

**Published:** 2024-10-14

**Authors:** Christiane Essoh, Yolande Hauck, Timothée Ouassa, Daouda Touré, Richmond Djatchi, Guillaume Yao Loukou, Simon-Pierre Assanvo N’Guetta, Gilles Vergnaud, Christine Pourcel

**Affiliations:** 1 Département de Biochimie-Génétique, UFR des Sciences Biologiques, Université Peleforo Gon Coulibaly (UPGC), Korhogo BP 1328, Côte d’Ivoire; daouda-toure@upgc.edu.ci; 2 Institute for Integrative Biology of the Cell (I2BC), CEA, CNRS, Université Paris-Saclay, 91198 Gif-sur-Yvette, France; yolande.hauck@i2bc.paris-saclay.fr (Y.H.); christine.pourcel@i2bc.paris-saclay.fr (C.P.); 3 Centre de Diagnostic et de Recherches sur le SIDA et les Autres Maladies Infectieuses (CeDReS), CHU de Treichville, Abidjan BPV 03, Côte d’Ivoire; timoth2@yahoo.com (T.O.); djatchirichmond@yahoo.fr (R.D.); 4 Laboratoire National de Santé Publique (LNSP), Abidjan BP 2403, Côte d’Ivoire; guillaumeloukou@yahoo.fr; 5 Laboratoire de Génétique, UFR Biosciences, Université Félix Houphouët-Boigny, Abidjan BPV 582, Côte d’Ivoire; nguettaewatty@yahoo.fr

**Keywords:** *Pseudomonas aeruginosa*, MLVA, multidrug-resistant, Côte d’Ivoire, genotyping, epidemiology

## Abstract

**Background/objectives:** *Pseudomonas aeruginosa* can cause community-acquired infections affecting various body sites. The present retrospective study investigated the genetic diversity of 173 isolates (166 clinical, 7 environmental) of *P. aeruginosa* collected from clinical pathology laboratories in Abidjan, Côte d’Ivoire (2001–2011). **Methods:** Multiple-Locus Variable Number of Tandem Repeats (VNTR) Analysis (MLVA) using 13 loci was applied to all isolates and compared to published MLVA data. The antibiotics status of the isolates was compiled when available and compared to published profiles. **Results:** Among 95 isolates analyzed for their antibiotics status, 14 displayed concerning resistance profiles: five multidrug-resistant (MDR) and nine extensively drug-resistant (XDR). MLVA typing revealed a high genetic diversity (>130 genotypes), with many genotypes represented by a single strain. Notably, thirteen clusters (≥4 related isolates) were observed. Some clusters displayed close genetic relatedness to isolates from France, Korea, and well-studied strains (ST560, LES and PA14). Comparative analysis suggested the presence of international high-risk MDR clones (CC233, CC111) in Côte d’Ivoire. Importantly, MLVA clustering revealed a close relationship of CC235-MDR strains with a locally identified cluster (group 9). **Conclusions:** These findings support MLVA as a reliable and cost-effective tool for low-resource settings, allowing the selection of relevant strains for future whole genome sequence analyses. This approach can improve outbreak investigations and public health interventions aimed at curbing MDR *P. aeruginosa* transmission within hospitals and at the national level.

## 1. Introduction

*Pseudomonas aeruginosa* is a highly versatile, Gram-negative bacterium capable of thriving in a wide range of environments, from soil to water and on medical surfaces. Its persistence is largely due to its capacity for adaptation and considerable metabolic flexibility, supported by a large genome (5.5–7 Mbp) [1]. This extensive genetic repertoire allows it to effectively respond to environmental stresses and adapt to diverse conditions, contributing to its ubiquity and its resilience in both clinical and non-clinical settings.

*P. aeruginosa* is a prominent opportunistic pathogen, causing a wide range of healthcare-associated infections (HAIs) in immunocompromised patients and chronic infection in cystic fibrosis patients [2]. Studies in Côte d’Ivoire highlighted its concerning prevalence, particularly in pediatric otitis and food sources contamination [3,4]. In addition, *P. aeruginosa* was reported in this country as one of the uropathogens with high levels of multidrug resistance [5].

The emergence of multidrug-resistant (MDR) *P. aeruginosa* strains represents a significant threat in healthcare settings, particularly for vulnerable patients [6]. This bacterium belongs to the ESKAPE group, notorious for its association with HAIs and rising antibiotic resistance, contributing significantly to mortality [7]. The extensive arsenal of resistance mechanisms employed by *P. aeruginosa*, including carbapenemase production, further complicates treatment options [8,9]. Due to this growing threat, *P. aeruginosa*, particularly carbapenem-resistant strains, were designated a high-priority pathogen in the latest WHO classification. Despite its significant health burden, the *P. aeruginosa* population structure remains poorly understood, particularly in developing countries [10]. Its identification relies essentially on phenotypic methods, including the characterization of the antibiotic resistance profiles for treatment purposes. This approach does not provide the comprehensive understanding necessary for efficient epidemiological surveillance of this clinically relevant pathogen. A few studies involving molecular typing were performed in Western and Central African countries in the context of international collaborative projects [10].

The population genetics of *P. aeruginosa* has been described as panmictic with the existence of some clonal complexes (CC) [11,12]. Drug resistance is a factor favoring the expansion of international CCs [13,14], but in conditions such as cystic fibrosis (CF) some antibiotics-susceptible clones were also observed, probably due to an adaptation of the strains to chronic carriage [15].

Diverse genotyping techniques have been employed to evaluate the genetic diversity of *P. aeruginosa* strains including random amplification of polymorphic DNA (RAPD), pulse field gel electrophoresis (PGFE), multilocus sequence typing (MLST), multiple locus VNTR (Variable Number of Tandem Repeats) analysis (MLVA) and whole genome sequencing associated with core-genome MLST (cgMLST) [16,17,18,19,20]. Among these techniques, MLST and MLVA provide the genotype under the form of a numeric code that can be stored in databases for easy international comparison. A large database of sequence types (ST) exists [21] but the technique remains too expensive for large studies to be performed. Whole genome sequencing approaches, which allow for deducing MLST types in silico at a lower cost than the original sequencing of individual loci, are not yet accessible at a significant scale in many developing countries. Interestingly, MLVA has been shown to predict MLST types at the CC level [22], providing a cost-effective and accessible alternative [23,24,25,26].

In this study, we used the MLVA genotyping method to investigate the genetic diversity of a large collection of clinical *P. aeruginosa* isolates from healthcare settings in Abidjan, Côte d’Ivoire. We also included environmental isolates to gain a more comprehensive understanding of the local *P. aeruginosa* population structure. We aimed to better understand the origin and spread of infections in order to implement control measures and improve their diagnosis, allowing for the rapid application of appropriate treatments in healthcare settings.

## 2. Materials and Methods

### 2.1. Bacterial Strains

A total of 173 *P. aeruginosa* isolates were collected from two medical institutions in Abidjan, Côte d’Ivoire. The «Centre de Diagnostic et de Recherche sur le SIDA et les autres maladies infectieuses» (CeDReS) contributed 114 isolates collected between 2001 and 2011 whereas the «Laboratoire National de Santé Publique» (LNSP) provided an additional 52 isolates collected during 2010–2011. One of these isolates, Ln50, was recovered from packaged drinking water. The clinical isolates originated from various specimens obtained from inpatients and outpatients treated at diverse healthcare facilities in Abidjan, primarily the Treichville Teaching Hospital. In addition, seven environmental isolates (Ev1 to Ev5, Ev7 and Ev8) from hospital sewage water in Abidjan, and two reference strains, PAO1 and PA14, were included in the analysis.

### 2.2. Isolation and Identification of P. aeruginosa

Bacteria were identified from the biological samples using conventional methods. Clinical specimens were cultured on Eosin Methylene Blue (EMB) agar medium. After overnight incubation at 37 °C, presumptive colonies were further analyzed by conventional biochemical tests such as glucose fermentation, oxidase, catalase and urea indole; ability to grow at 42 °C; and ability to produce characteristic pigmentations on cetrimide agar. Subsequently, King A and King B media were inoculated to differentiate *P. aeruginosa*, which grows and produce pigments on both media, from other *Pseudomonas* species, which only grow on King B.

For the isolation of *P. aeruginosa* from environmental samples, filtrated water was enriched in Brain Heart Infusion (BHI) medium, then 100 µL were streaked onto cetrimide agar and plates were incubated at 42 °C for 24 h. Suspect bacterial colonies with green pigmentation were used for biochemical tests and Gram coloration staining.

### 2.3. Antibiotic Susceptibility Testing

Antimicrobial susceptibility was determined using the Kirby–Bauer disk diffusion method on Mueller–Hinton agar [27] and interpreted with clinical breakpoints from the Antibiogram Committee of the French Society of Microbiology and EUCAST (CA-SFM/EUCAST) revisited for 2020 [28]. Eleven antimicrobial agents were tested using antibiotic disks (Bio-Rad, Marnes-la-Coquette, France) piperacillin (PIP 30 µg), ticarcillin (TIC 75 µg), cefsulodin (CFS 30 µg), ceftazidime (CAZ 30 µg), aztreonam (ATM 30 µg), imipenem (IMP 10 µg), amikacin (AMK 30 µg), tobramycin (TOB 10 µg), gentamicin (GEN 10 µg), netilmicin (NET 10 µg), and ciprofloxacin (CIP 5 µg). Cefsulodin is not recommended for testing by current guidelines but was used in CeDReS until 2015.

The WHONET 5.6 software from the World Health Organization was employed to analyze the antibiotic resistance profiles and categorize the isolates based on established criteria. This software identified various phenotypes, including multidrug-resistant (MDR), extensively drug resistant (XDR), and non-MDR isolates.

### 2.4. DNA Purification

DNA extraction was performed using the standard phenol-chloroform method [29] with minor modifications. Briefly, bacterial colonies were suspended in TE buffer (Tris-HCL 10 mM, EDTA 1 mM pH 8.0) and the mixture was treated with 0.2 µg/mL RNase A to remove RNA contamination. After 15 min at room temperature, the bacterial cells were lysed with one volume of 2× lysis buffer containing 20 mM Tris-HCl (pH 8), 20 mM EDTA, 1% sodium dodecyl sulfate (SDS), 20 mM NaCl, followed by incubation with 0.5 µg/mL proteinase K at 55 °C for 2 h.

DNA purification was carried out through three consecutive extraction cycles using phenol, phenol-chloroform (1/1), and chloroform. Each extraction step was followed by centrifugation at 13,000 rpm for 10 min. DNA precipitation was performed with two volumes of absolute ethanol and 200 mM NaCl, final concentration. After centrifugation, the DNA pellet was washed twice with 70% ethanol to remove residual contaminants. Following drying at room temperature, the purified DNA was resuspended in TE buffer. The DNA concentration was quantified using a NanoDrop^®^ ND-1000 spectrophotometer (Labtech, Palaiseau, France).

### 2.5. MLVA Genotyping

The MLVA scheme was performed using 13 VNTRs loci and the PCR amplification protocol as described by Vu-Thien et al. [17]. The MLVA-13 genotype is expressed as its allelic profile corresponding to the number of repeats at each VNTR in the order ms77, ms127, ms142, ms172, ms211, ms212, ms213, ms214, ms215, ms216, ms217, ms222, and ms223. The genotype of PAO1 deduced from its genomic sequence (genome nucleotide sequence accession NC_002516.1) is 4 8 7 12 5 9 5 3 4 3 2 2 4. The *P. aeruginosa* MLVA-13 assay is easy to run owing to the large repeat unit of most VNTR loci in this species [17]. Nine among the 13 loci used in MLVA-13 have repeat units with a length above 100 bp. Figure S1 shows an agarose gel electrophoresis of PCR amplicons from ms216 (upper part) and ms223 (lower part), derived from 30 *P. aeruginosa* isolates (lanes labelled 1 to 30), and the reference strain PAO1 (lanes labelled ‘R’).

The BioNumerics software package 7.6.3 (Applied Maths, St-Martens-Latem, Belgium) was used to perform clustering analyses with the categorical coefficient (also called Hamming’s distance) and the Unweighted Pair Group Method with Arithmetic mean (UPGMA) algorithm, producing a dendrogram. Clusters were defined as groups of isolates sharing at least seven VNTR alleles defining a 60–100% similarity (60% cut-off) as previously conducted in a study of antibiotic resistant *P. aeruginosa* strains [30]. Additionally, a minimum spanning tree (MST) analysis was performed, allowing the observation of clusters in a more condensed form.

## 3. Results

### 3.1. Distribution of P. aeruginosa Isolates by Source

Among the 173 *P. aeruginosa* isolates (including seven environmental isolates), pus samples emerged as the most frequent source, accounting for half of the isolates (Table 1). This category encompassed ear pus, abscesses, operative pus, and pus swab samples, with ear pus being the most prevalent type, representing 10% of the isolates. This is in agreement with previous reports highlighting *P. aeruginosa* as a significant etiologic agent in otitis media cases in Côte d’Ivoire, particularly in children, and more globally in sub-Saharan Africa [3,31].

Blood and urine cultures yielded *P. aeruginosa* in 10.4% and 11.0% of isolates, respectively. Other sources, including pleural fluid, bronchial aspirates, and cerebrospinal fluid, collectively contributed 6.4% of the isolates. Environmental isolates from wastewater represented 4% of the total collection. Unspecified biological samples from the LNSP collection represented the second most common source (22.5%).

### 3.2. Antibiotic Resistance Profiles

**Figure 1:**
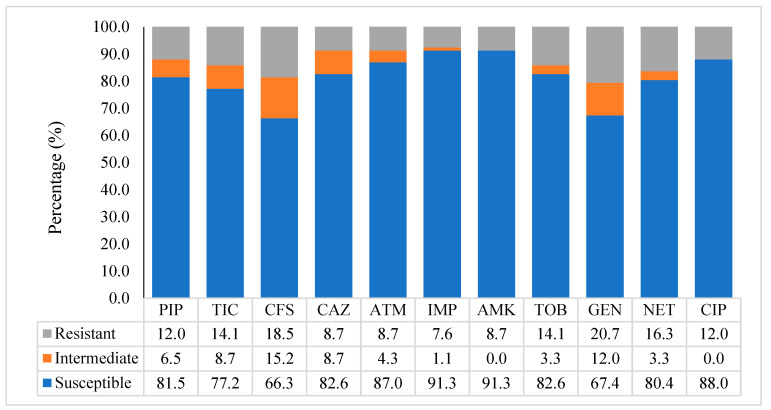
Antimicrobial resistance pattern of *P. aeruginosa* isolates from CeDReS collected in 2001–2011; piperacillin (PIP), ticarcillin (TIC), cefsulodin (CFS), ceftazidim (CAZ), aztreonam (ATM), imipenem (IPM), amikacin (AMK), tobramycin (TOB), gentamicin (GEN), netilmicin (NET), and ciprofloxacin (CIP).

Antibiotic (Ab) susceptibility testing was performed on 95 *P. aeruginosa* isolates, primarily from the CeDReS collection spanning 2001–2011 (*n* = 93). Two isolates from the LNSP collection were included. A full Ab-resistance profile was not available for the remaining LNSP strains, which were therefore excluded from this analysis. Figure 1 illustrates the antibiotic resistance levels in the CeDReS isolates. *P. aeruginosa* generally exhibited sensitivity to all tested antibiotics. Among *β*-lactam antibiotics, imipenem displayed the highest effectiveness (91.3% sensitivity), followed by aztreonam (87.0%), ceftazidime (82.6%), piperacillin (81.5%), ticarcillin (77.2%), and cefsulodin (66.5%). Aminoglycoside sensitivities varied, with amikacin being the most effective (91.3%), followed by tobramycin (82.6%), netilmicin (80.4%), and gentamicin (67.4%). Ciprofloxacin, the sole tested quinolone, had a sensitivity rate of 88.0%.

Utilizing WHONET 5.6 software, 14 drug-resistant isolates were identified: five multidrug-resistant (MDR) and nine potentially extensively drug-resistant (XDR), including both isolates from the LNSP collection. Most of these resistant isolates were collected in 2010 and 2011.

### 3.3. Genetic Diversity of P. aeruginosa Isolates

**Figure 2:**
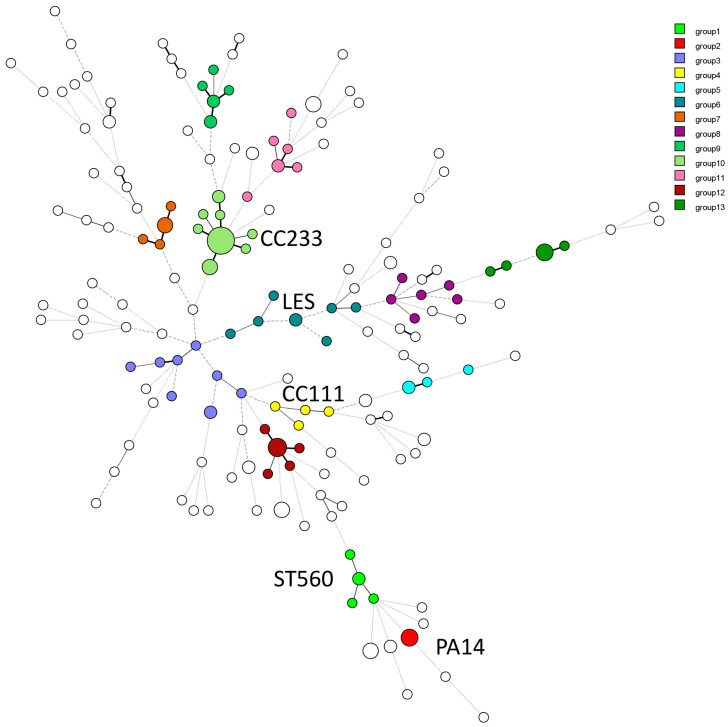
Minimum Spanning Tree (MST) analysis based on the MLVA-13 profile of 173 *P. aeruginosa* isolates collected in Côte d’Ivoire compared with 36 strains of various origins. Each circle represents a genotype, and the circle size is proportional to the number of samples within that specific genotype. Branch lengths above three are dashed. The 13 clusters identified by UPGMA (Figure S2) and comprising 82 isolates from Côte d’Ivoire are colored as indicated. The other isolates are singletons or belong to smaller clusters.

The MLVA-13 profiles of the 173 isolates from the present report, plus 36 genetically diverse control strains originating from various countries, produced 159 genotypes of which 130 were specific to Abidjan’s isolates. A cut-off value of 60% genetic similarity defined 80 clusters, with 13 clusters containing at least four isolates (highlighted by color in Figure 2 and represented as a dendrogram in Figure S2).

The largest cluster (group 10) comprised 19 clinical isolates and environmental isolate Ev5. One group 10 genotype was identical to the MLVA genotype of French strains FrCI21 and PAC1-17 [30,32]. These strains were previously shown to belong to MLST CC233, known to harbor the VIM-2 metallo-*β*-lactamase gene [33]. Seven of the eight XDR isolates identified in this study belonged to group 10.

Other clusters exhibited genetic relatedness to well-characterized CCs. Groups 1, 2, 4, and 6 included isolates with close similarity to ST560, PA14, CC111, and LES clones, respectively. Eight isolates clustered with the frequently isolated British strain LESB58, while three each matched the PA14 and CC111 strains. The latter are known for harboring MDR-associated antibiotic resistance cassettes [32]. Four isolates were closely associated with clone ST560. In addition, one isolate exhibited near-complete identity with PAO1, with a single difference at locus ms212. Interestingly, 22 clusters encompassing non-MDR isolates displayed similarities with strains from French cystic fibrosis (CF) patients. Groups 8, 9, 11, 12, and 13 were the most prevalent in this category. In contrast, isolates within groups 3, 5, 7, and 9 lacked close genetic matches with strains from other countries included in the study. As expected, due to their diverse origins, the environmental isolates were genetically different. Ev5 and Ev7 showed similarities with specific clinical strains.

### 3.4. Genetic Relationships with International Strains

**Figure 3:**
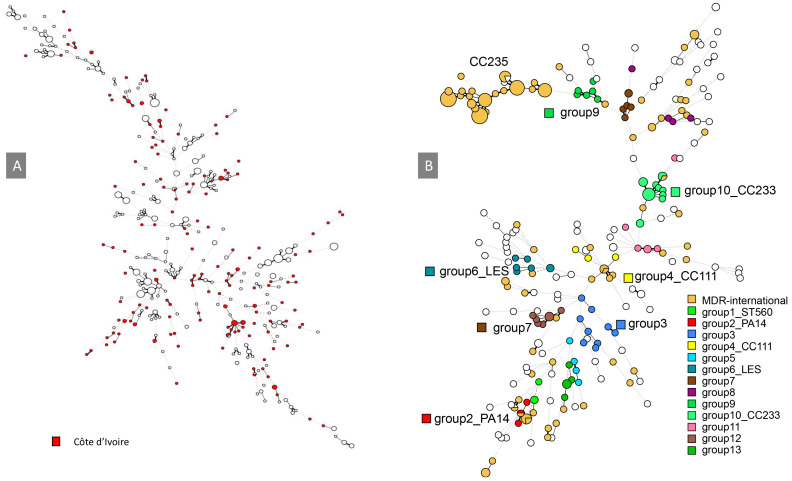
MSTs showing the global distribution of *P. aeruginosa* isolates, with (**A**) 173 isolates of Ivorian origin in red and 408 cystic fibrosis isolates from Europe in open circles. (**B**) Ivorian isolates with 127 international-MDR strains shown in orange. The circle size is proportional to the number of samples within that specific genotype.

The online MLVA database from the University Paris-Saclay, France, was used to compare the genetic relationships between Ivorian isolates and strains from other countries, primarily from Europe and Korea and was updated with the present collection (Table S1) [34]. The Minimal Spanning Tree (MST) clustering method facilitated the comparison of numerous genetic profiles. The clustering analysis demonstrated that most Ivorian isolates possessed unique genotypes, scattered throughout the tree (Figure 2). A comparison with *P. aeruginosa* strains associated with cystic fibrosis (CF) in France showed a more homogenous bacterial population, with most CF strains clustering together (Figure 3A). Except for the strains clustering with CC111 (group 4) and CC233 (group 10), Ivorian isolates displayed a distinct genetic signature when compared to a global collection of MDR strains (Figure 3B). Interestingly, local group 9 was in a position in the clustering analysis close to CC235-MDR type isolates, suggesting a possible relationship between these two complexes. These findings highlight the presence of both locally unique and geographically dispersed strains within the country.

## 4. Discussion

The present retrospective study investigated the global genetic diversity of *P. aeruginosa* isolates in Côte d’Ivoire through analysis of a large collection spanning a decade (2001–2011). MLVA typing revealed high genetic diversity among the Ivorian *P. aeruginosa* isolates, with one half possessing unique genotypes and clustering of the other half. This finding supports the notion of a non-clonal population structure for *P. aeruginosa*, with the presence of a few epidemic strains as previously reported [12]. Isolates showing similarities with reference strains PAO1, LES, and PA14 were identified, in agreement with a study conducted in Kenya [35]. Clustering allowed for the definition of groups that were compared to those observed in other studies performed by our team and others.

Among the Ab-resistant strains, a few displayed genetic similarities with strains from other countries. Group 10, the most prevalent cluster, comprised carbapenem-resistant XDR isolates from CeDReS, alongside XDR isolates from LNSP and several others with unrecorded antibiotic profiles, including environmental isolate Ev5. These isolates exhibited resistance to various antibiotic classes but remained susceptible to monobactams, mirroring the French strain FrCI21 [32]. Genetic analysis linked them to international MDR strains of serotype O6 harboring VIM-2 metallo-*β*-lactamase (MBL) genes, characteristics consistent with the previously reported ST233 clone in Ivorian clinical isolates [36]. This suggests that group 10 isolates belong to CC233, potentially explaining their rapid emergence and dissemination within the hospital environment. Members of CC233 are considered “high-risk clones” with a global presence in Europe, Asia, and Africa [33]. Based on the antibiotic profiles from the CeDReS collection spanning a decade, it appears that CC233 was already present in Côte d’Ivoire a few years after the discovery of the ST233 clone in 2006 from a Norwegian patient infected in a hospital in Ghana, a country adjacent to Côte d’Ivoire [37], suggesting that this clone is endemic in this region. Additionally, isolates belonging to CC111, typically associated with MDR phenotypes due to VIM-2 production [33], were identified. However, unlike pandemic CC111 strains, these isolates were susceptible to most antibiotics, as previously reported [10,38]. The authors suggested that the spread of certain high-risk clones might be independent of antimicrobial resistance and linked to other factors like host adaptation, virulence, or biofilm formation.

Group 9 strains clustered nearby CC235, a widespread pandemic “high-risk clone” associated with carbapenem resistance through various MBL productions [33,39]. *P. aeruginosa* isolates belonging to CC235 have been reported in Côte d’Ivoire [10] and in Indonesia [40]. While CC235 isolates have not been directly observed in this study, the group 9 clustering relationship suggests a possible link of this local group with CC235. Whole genome sequencing of group 9 isolates will allow for testing this prediction.

Carbapenem resistance has emerged as an escalating threat to public health, particularly in regions with limited resources and restricted access to healthcare. Although the present study observed a low prevalence of carbapenem resistance among the CeDReS decade-old collection, a concerning trend occurred with several isolates exhibiting an XDR phenotype, primarily isolated between 2010 and 2011. The observed increase in prevalence over a short period suggests the emergence of an epidemic clone within Ivorian hospitals, which emphasizes the importance of MDR surveillance programs. Unfortunately, limitations in patient hospitalization data hindered a deeper understanding of factors contributing to the dissemination of the highly resistant isolates. Enhanced awareness among healthcare personnel regarding the collection of comprehensive clinical and epidemiological data is crucial for effective monitoring and control.

An important observation is the genetic similarities between some hospital wastewater and clinical isolates, suggesting the existence of environmental reservoirs for *P. aeruginosa* that are contributing to patient infections [41]. Indeed, *P. aeruginosa* exhibits a panmictic population structure in which environmental and clinical strains are indistinguishable [42]. The isolation of Ev5, belonging to group 10 (high-risk CC233), strengthens the possibility of hospital environmental contamination. This finding is in agreement with the high prevalence of group 10 isolates, potentially reflecting dissemination within the hospital. Effective control of *P. aeruginosa*, especially its MDR variants, necessitates rigorous epidemiological surveillance of circulating clones within healthcare settings.

Interestingly, isolate LN50, which is genetically close to ST560 strains, was detected in a sample of commercially distributed drinking water in Côte d’Ivoire. ST560 is an intercontinental clone that has been previously observed in animals [43], during intestinal colonization [44], associated with human outbreaks [45], and in environmental samples [46]. The remarkable metabolic plasticity of *P. aeruginosa* may explain its capacity to survive in packaged drinking water. A previous study reported the presence of *P. aeruginosa* in food items, such as fresh and smoked meats and fish, which are widely consumed in Côte d’Ivoire [4], potentially leading to foodborne infections. These findings underline the importance of considering environmental and dietary factors in the study of *P. aeruginosa*-caused infections.

## 5. Conclusions

The treatment of patients suffering from infections in health care institutions requires a precise identification of the bacterial species and of the antibiotic susceptibility of the isolates. It is also important to investigate the genetic diversity of the strains in order to evaluate the risk for transmission of drug-resistant clones, and to implement control measures. We believe that genotyping of *P. aeruginosa* strains could be part of the diagnostic procedure provided that simple and cost-effective methods exist. Our results show that the use of MLVA can provide at a reasonable cost, important information on strains circulating in hospitals.

This relatively cost-effective technique offers accurate strain characterization for epidemiological investigations. Due to its affordability and accessibility for low-resource laboratories, MLVA can be readily implemented for routine monitoring of *P. aeruginosa* strains and identification of potential infectious sources.

In the era of genomic pathogen surveillance, a practical approach for low-income countries involves utilizing MLVA data to identify prevalent clonal groups. Representative strains from these clusters can then be selected for complete genome sequencing, providing a deeper understanding of the circulating *P. aeruginosa* population. This combined strategy offers a cost-effective and informative approach for countries with limited resources.

## Figures and Tables

**Table 1:** Distribution of *P. aeruginosa* isolates according to biological samples.

Biological Samples		CeDReS		LNSP		Total (%)	
		*n* = 114	%	*n* = 59	%		
Clinic	Pus	40	35.1	0	0.0	40	23.1
	Middle ear	16	14.0	1	1.7	17	9.8
	Swab	8	7.0	1	1.7	9	5.2
	Wound	2	1.8	7	11.9	9	5.2
	Abscess	4	3.5	0	0.0	4	2.3
	Blood	16	14.0	2	3.4	18	10.4
	Urine	18	15.8	1	1.7	19	11.0
	Other	8	7.0	3	5.1	11	6.4
	Not specified	2	1.8	37	62.7	39	22.5
Environment	Waste water	0	0.0	7	11.9	7	4.0

## Data Availability

The data presented in this study are available in Supplementary File Table S1 and further inquiries can be directed to corresponding author CE.

## Supplementary Materials

The following supporting information can be downloaded at: https://www.mdpi.com/article/10.3390/diagnostics14202284/s1, Figure S1: Agarose gel electrophoresis of PCR amplicons from two VNTRs ms216 and ms223 in 30 strains and the reference strain PAO1; Figure S2: Genetic diversity of *P. aeruginosa* strains collected in Abidjan, Côte d’Ivoire; Table S1: MLVA data.
