# Supplementary material for: Molecular Typing of Pseudomonas aeruginosa Isolates Collected in Abidjan Hospitals (Côte d’Ivoire) Using the Multiple-Locus Variable Number of Tandem Repeats Method
Source: Diagnostics (Basel). 2024 Oct 14;14(20):2284. doi: 10.3390/diagnostics14202284 (PMC11506784; doi:10.3390/diagnostics14202284)
Supplement: Supplementary file 1 [file diagnostics-14-02284-s001.zip › Figure S1.pdf]

Figure S1: Agarose gel electrophoresis of PCR amplicons from two VNTRs, ms216 (upper) and ms223 (lower), derived from 30 *P. aeruginosa* isolates, including the reference strain PAO1 (lanes labelled 'R'). Isolates are labeled from lane 1 to 30, with each group of six samples, including the reference strain (lane R), separated by the 100bp ladder molecular weight marker (M). The scale on the left indicates the size of each band (base-pairs). Alleles of 543 bp (3U) and 454 bp (4U) are expected in the reference strain (lane R) for ms216 and ms223 respectively. Ms216 has a repeat unit of 113 bp and ms223 of 106 bp so the observed alleles can be readily converted to repeat copy numbers by visual examination.

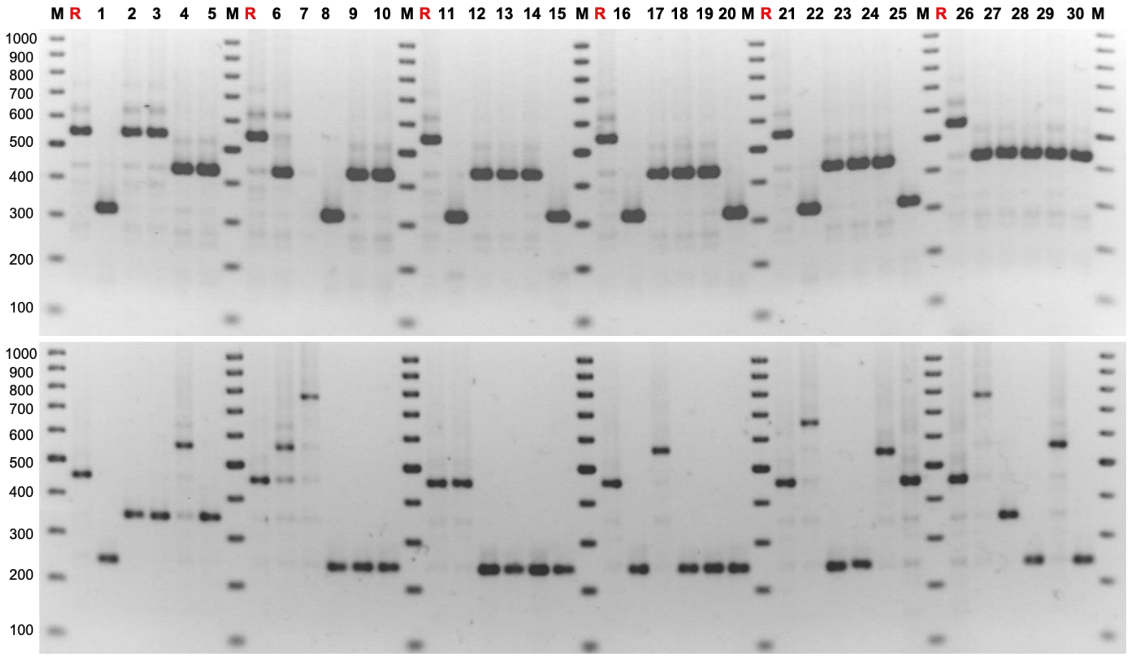

Figure S1 Essoh et al. 2024, Diagnostics
