# Supplementary material for: Molecular Typing of Pseudomonas aeruginosa Isolates Collected in Abidjan Hospitals (Côte d’Ivoire) Using the Multiple-Locus Variable Number of Tandem Repeats Method
Source: Diagnostics (Basel). 2024 Oct 14;14(20):2284. doi: 10.3390/diagnostics14202284 (PMC11506784; doi:10.3390/diagnostics14202284)
Supplement: Supplementary file 1 [file diagnostics-14-02284-s001.zip › Figure S2.pdf]

Figure S2: Genetic diversity of 173 *P. aeruginosa* strains collected in Abidjan, Côte d'Ivoire and 36 public datasets (same strain collection as used in Figure 2). The dendrogram was generated using BioNumerics based on MLVA-13 data. Thirteen groups defined with a similarity threshold of 60% and comprising at least four isolates/strains are indicated by colors. Healthcare facilities are color-coded.

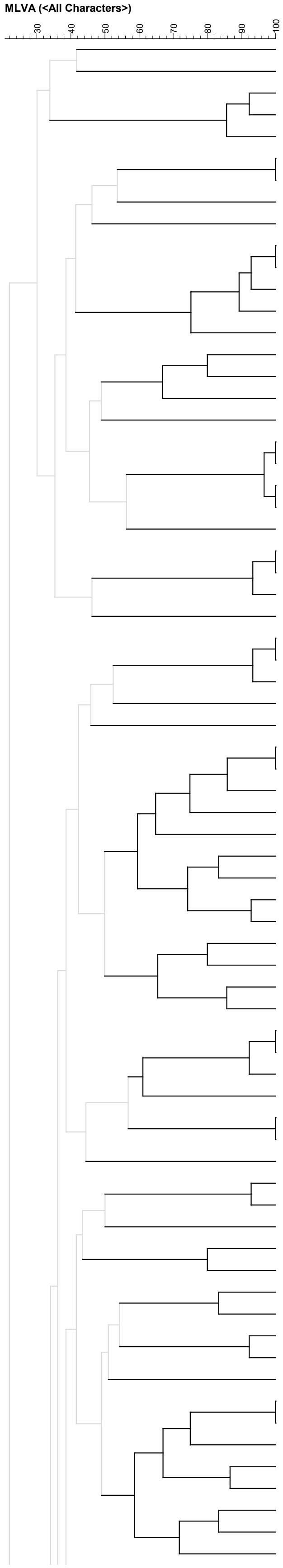

Group	Strains	Establishment	Date	Source	ATB profile
	cd21-52	CeDres	26.10.2009	Pus	Not MDR
	Ln1	LNSP	01.10.2010	Wound	ND
	cd29-24	CeDres	03.03.2011	Pus	MDR
	Papcy022	Percy		Urine	
	cd25-47	CeDres	12.07.2010	Catheter	XDR
	Ln38	LNSP	10.10.2011	Clinical	ND
	PcyII-40	Percy		CTB	
	Ln40	LNSP	10.10.2011	Wound	Not MDR
	cd09-31	CeDres	14.07.2003	Blood	Not MDR
group1	Ln4	LNSP	01.07.2010	Clinical	ND
group1	Ln8	LNSP	01.10.2010	Clinical	ND
group1	PAC1-20	Besançon	23.01.2007	CF	
group1	Ln50	LNSP	10.09.2010	Drink Water	ND
group1	K16	Korea		Urine	
	cd03-81	CeDres	09.01.2002	Pus	Not MDR
	Tr172	Trousseau	12.07.2006	CF	
	Ln13	LNSP	10.09.2010	Wound	Not MDR
	cd26-14	CeDres	18.08.2010	Blood	MDR
group2	PAC9-12	Toulouse	15.02.2007	CF	
group2	PA14	Genbank			
group2	cd27-28	CeDres	29.09.2010	Swab sample	Not MDR
group2	Ln45	LNSP	10.10.2011	Clinical	ND
	cd03-48	CeDres	16.11.01	Blood	Not MDR
	Ln46	LNSP	10.10.2011	Clinical	ND
	Fr10i	Narbonne	25.09.2010	Urine	
	Ln24	LNSP	05.10.2011	Clinical	ND
	cd23-80	CeDres	23.04.2010	Pus	ND
	cd25-70	CeDres	24.08.2010	Swab sample	Not MDR
	cd25-71	CeDres	27.07.2010	Catheter	MDR
	cd25-72	CeDres	27.07.2010	Swab sample	Not MDR
	cd29-20	CeDres	18.02.2011	Abscess	Not MDR
	cd06-72	CeDres	26.12.2002	Middle ear	Not MDR
group3	cd27-20	CeDres	22.09.2010	Urine	Not MDR
group3	Ln20	LNSP	01.10.2011	Swab sample	Not MDR
group3	Ln39	LNSP	10.10.2011	Clinical	ND
group3	Ln42	LNSP	10.10.2011	Wound	MDR
group3	cd27-21	CeDres	22.09.10	Pleural exudate	Not MDR
group3	cd09-65	CeDres	15.09.2003	Pus	Not MDR
group3	cd23-60	CeDres	02.04.2010	Blood	Not MDR
group3	Ln36	LNSP	10.10.2011	Clinical	ND
group3	Ln37	LNSP	10.10.2011	Clinical	ND
group4	Ev4	LNSP	05.10.2011	waste water	ND
group4	K8	Korea		Sputum	
group4	Ln9	LNSP	01.04.2010	Clinical	ND
group4	Ln10	LNSP	01.102010	Clinical	ND
group5	cd03-35	CeDres	04.10.2001	Pus	Not MDR
group5	cd04-38	CeDres	01.03.2002	Pus	Not MDR
group5	cd23-27	CeDres	04.03.2010	Pus	Not MDR
group5	cd03-28	CeDres	27.09.2001	Pus	Not MDR
	cd04-62	CeDres	04.04.2002	Blood	Not MDR
	cd04-63	CeDres	05.04.2002	Blood	Not MDR
	cd30-21	CeDres	17.06.2011	Abscess	MDR
	cd29-55	CeDres	18.03.2011	Urine	Not MDR
	cd29-18	CeDres	29.04.2011	Swab sample	Not MDR
	cd10-50	CeDres	03.02.2004	Urine	Not MDR
	cd10-11	CeDres	03.11.2003	Middle ear	Not MDR
	PAC5-10	Lille	07.02.2007	CF	
	cd23-44	CeDres	18.03.2010	Bronchial Aspirate	Not MDR
	cd25-40	CeDres	28.06.2010	Pus	Not MDR
	cd26-74	CeDres	03.09.2010	Catheter	Not MDR
	cd02-60	CeDres	08.06.2001	Middle ear	Not MDR
	cd30-33	CeDres	25.07.2011	Pleural exudate	Not MDR
group6	cd12-17	CeDres	22.09.2004	Pus	Not MDR
group6	cd12-64	CeDres	12.01.2005	Pus	ND
group6	LES	Genbank			
group6	cd09-71	CeDres	24.09.2003	Pus	ND
group6	cd29-78	CeDres	29.04.2011	Pus	MDR
group6	cd08-37	CeDres	07.05.2003	Blood	Not MDR
group6	cd10-24	CeDres	21.11.2003	Middle ear	MDR
group6	cd23-47	CeDres	18.03.2010	Blood	Not MDR

ST560

PA14

CC111

LES

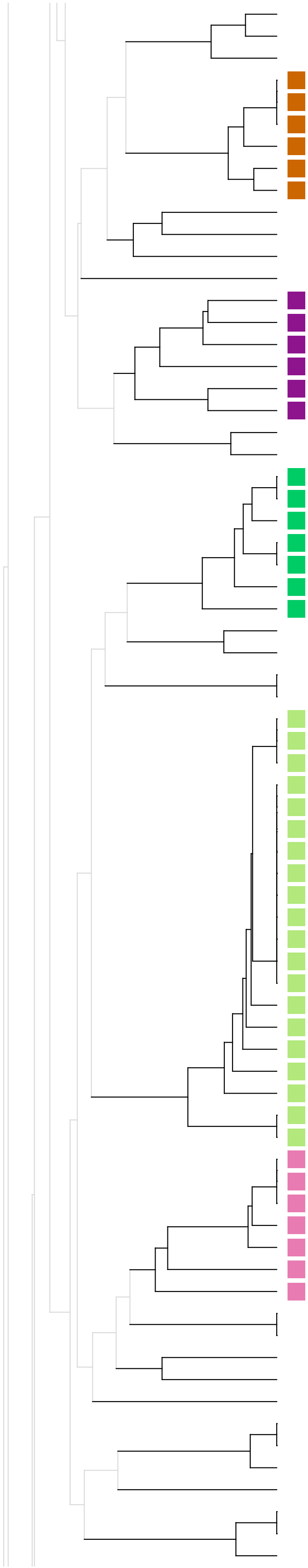

	cd23-81	CeDres	23.04.2010	Pus	Not MDR
	Ln21	LNSP	01.10.2011	Bronchial Aspirate	Not MDR
	PAC5-11	Lille	23.02.2007	CF	
group7	cd16-15	CeDres	12.06.2008	Urine	Not MDR
group7	Ln52	LNSP	20.10.2010	Wound	ND
group7	cd16-16	CeDres	12.06.2008	Urine	Not MDR
group7	Ln5	LNSP	01.02.2011	Clinical	ND
group7	cd24-28	CeDres	14.05.2010	Pus	Not MDR
group7	cd24-29	CeDres	14.05.2010	Pus	Not MDR
	cd26-71	CeDres	24.08.2010	Pleural exudate	MDR
	PAC10-14	Nantes	10.01.2020	CF	
	cd10-57	CeDres	12.02.2004	Pus	ND
	cd27-19	CeDres	22.09.2010	Swab sample	Not MDR
group8	cd26-50	CeDres	24.08.2010	Pus	Not MDR
group8	PAC7-6	Montpellier	15.03.2007	CF	
group8	Ln43	LNSP	10.10.2011	Clinical	ND
group8	cd05-21	CeDres	07.06.2002	Middle ear	ND
group8	Ln41	LNSP	10.10.2011	Otitis	Not MDR
group8	K35	Korea		Wound swab	
	cd29-22	CeDres	03.03.2011	Bronchial Aspirate	Not MDR
	cd29-23	CeDres	03.03.2011	Abscess	MDR
group9	cd08-40	CeDres	12.05.2003	Blood	Not MDR
group9	Ln6	LNSP	10.01.2010	Clinical	ND
group9	cd04-45	CeDres	15.03.2002	Pus	Not MDR
group9	cd26-73	CeDres	03.09.2010	Swab sample	Not MDR
group9	PAC6-16	Cochin/SVP	14.02.2007	CF	
group9	cd08-27	CeDres	28.04.2003	Middle ear	Not MDR
group9	Ln48	LNSP	10.10.2011	Clinical	ND
	cd04-12	CeDres	25.01.2002	Pus	Not MDR
	PAC8-5	R-Debré	01.02.2007	CF	
	cd26-72	CeDres	03.09.2010	Blood	ND
	Tr191	Trousseau	05.10.2006	CF	
group10	cd25-38	CeDres	28.06.2010	Urine	XDR
group10	cd26-38	CeDres	18.08.2010	Pus	XDR
group10	cd28-03	CeDres	27.11.2010	Cerebrospinal fluid	XDR
group10	cd26-13	CeDres	18.08.2010	Pus	XDR
group10	cd27-30	CeDres	29.09.2010	Urine	XDR
group10	Ln23	LNSP	05.10.2011	Clinical	ND
group10	Ln25	LNSP	05.10.2011	blood	XDR
group10	Ln26	LNSP	05.10.2011	Clinical	ND
group10	Ln27	LNSP	05.10.2011	Clinical	ND
group10	Ln28	LNSP	05.10.2011	Clinical	ND
group10	Ln31	LNSP	05.10.2011	Blood	XDR
group10	Ln32	LNSP	10.10.2011	Clinical	ND
group10	FrCl21	Percy		Urine	
group10	Ln34	LNSP	05.11.2011	Clinical	ND
group10	Ln11	LNSP	01.10.2010	Clinical	ND
group10	Ln16	LNSP	01.10.2011	Clinical	ND
group10	Ln47	LNSP	10.10.2011	Clinical	ND
group10	PAC1-17	Besançon	16.01.2007	CF	
group10	Ev5	LNSP	05.10.2011	waste water	ND
group10	Ln22	LNSP	05.10.2011	Clinical	ND
group11	Ln17	LNSP	01.10.2011	Clinical	ND
group11	Ln29	LNSP	01.10.2011	Clinical	Not MDR
group11	Ln33	LNSP	05.11.2011	Clinical	ND
group11	cd30-61	CeDres	20.10.2011	Urine	Not MDR
group11	PAC6-9	Cochin/SVP	09.02.2007	CF	
group11	cd10-29	CeDres	12.12.2003	Burn	ND
group11	cd04-69	CeDres	12.04.2002	Middle ear	Not MDR
	cd02-44	CeDres	16.05.2001	Middle ear	MDR
	cd05-1	CeDres	30.04.2002	Pus	ND
	Ln7	LNSP	01.05.2011	Clinical	ND
	PAC9-8	Toulouse	07.02.2007	CF	
	Ln30	LNSP	05.10.2011	Pleural exudate	Not MDR
	cd26-12	CeDres	18.08.2010	Catheter	MDR
	cd26-75	CeDres	03.09.2010	Swab sample	Not MDR
	Tr220	Trousseau	18.07.2007	CF	
	cd23-17	CeDres	22.02.2010	Pus	Not MDR
	cd06-12	CeDres	11.10.2002	Pus	Not MDR
	Ev7	LNSP	05.10.2010	Waste water	ND
	PAC9-4	Toulouse	08.02.2007	CF	

CC235

VIM2 cluster
CC233

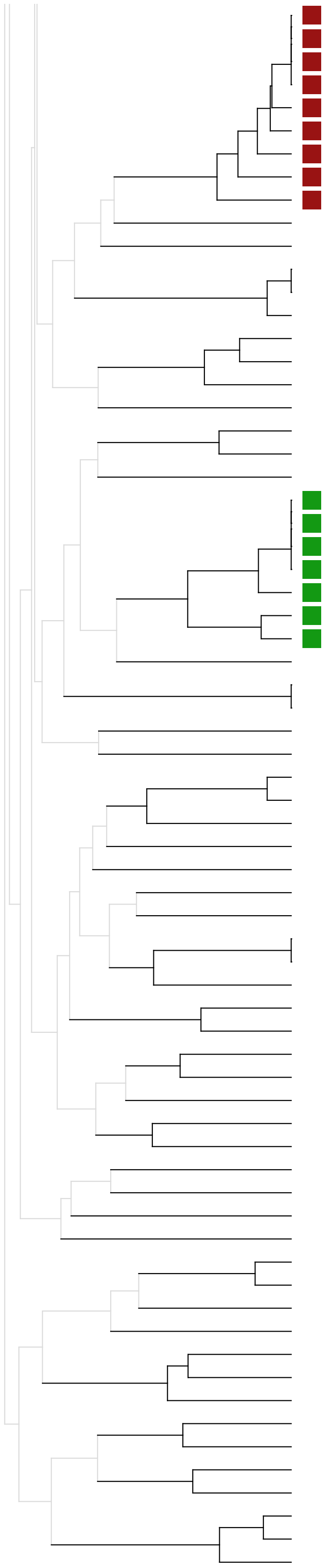

group12	cd27-29	CeDres	29.09.2010	Swab sample	Not MDR
group12	Ln15	LNSP	01.11.2011	Clinical	ND
group12	Ln18	LNSP	01.11.2011	Clinical	ND
group12	Ln19	LNSP	01.10.2011	Clinical	ND
group12	Ln44	LNSP	10.10.2011	Clinical	ND
group12	PAC2-3	Trousseau	07.02.2007	CF	
group12	Ln14	LNSP	01.10.2011	Clinical	ND
group12	Ln35	LNSP	10.10.2011	Clinical	ND
group12	cd09-4	CeDres	24.06.2003	Middle ear	Not MDR
	cd09-51	CeDres	18.08.2003	Middle ear	Not MDR
	cd10-43	CeDres	19.01.2004	Middle ear	Not MDR
	cd17-2	CeDres	11.09.2008	Pus	Not MDR
	cd17-47	CeDres	28.08.2008	Blood	ND
	PAC5-1	Lille	19.02.2007	CF	
	Ln12	LNSP	01.10.2010	Urine	Not MDR
	PAO1	Genbank			
	cd02-63	CeDres	14.06.2001	Blood	Not MDR
	cd02-62	CeDres	11.06.2001	Pus	ND
	cd25-39	CeDres	28.06.2010	Blood	Not MDR
	PAC3-16	Caen	27.10.2006	CF	
	Ev2	LNSP	05.10.2011	waste water	ND
group13	cd05-85	CeDres	18.05.2002	Middle ear	ND
group13	cd06-33	CeDres	14.11.2002	Pus	Not MDR
group13	cd06-61	CeDres	20.12.2002	Pus	Not MDR
group13	cd11-77	CeDres	30.08.2004	Pus	Not MDR
group13	PAC8-17	R-Debré	20.02.2007	CF	
group13	Ln49	LNSP	01.09.2010	Burn	MDR
group13	Ln3	LNSP	01.09.2010	Clinical	ND
	cd28-37	CeDres	14.01.2011	Clinical	ND
	cd30-37	CeDres	25.07.2011	Urine	MDR
	cd30-62	CeDres	28.09.2011	Pus	MDR
	cd29-04	CeDres	11.02.2011	Clinical	ND
	K14	Korea		Urine	
	cd29-49	CeDres	18.03.2011	Abscess	Not MDR
	Tr95	Trousseau	04.01.2006	CF	
	cd12-13	CeDres	27.09.2004	Pus	ND
	cd14-26	CeDres	05.09.2005	Pus	ND
	Ev1	LNSP	05.10.2011	waste water	ND
	Ev3	LNSP	05.10.2011	waste water	ND
	K19	Korea		Urine	
	Ln51	LNSP	16.09.2010	Wound	Not MDR
	PAC3-12	Caen	23.10.2006	CF	
	cd24-80	CeDres	03.06.2010	Swab sample	Not MDR
	cd09-81	CeDres	05.10.2003	Middle ear	Not MDR
	cd23-43	CeDres	11.03.2010	Pus	ND
	cd23-26	CeDres	04.03.2010	Pus	Not MDR
	PAC8-20	R-Debré	06.03.2007	CF	
	cd25-16	CeDres	17.06.2010	Pleural exudate	Not MDR
	cd06-65	CeDres	20.12.2002	Middle ear	Not MDR
	PAC4-3	Clermont-Ferrand	10.10.2007	CF	
	cd24-30	CeDres	14.05.2010	Blood	Not MDR
	cd25-37	CeDres	28.06.2010	Pleural exudate	Not MDR
	cd17-30	CeDres	19.08.2008	Urine	Not MDR
	cd28-35	CeDres	05.01.2011	Urine	Not MDR
	cd07-15	CeDres	04.02.2003	Middle ear	Not MDR
	cd08-35	CeDres	06.05.2003	Middle ear	MDR
	cd27-21	CeDres	22.09.2010	Pleural exudate	Not MDR
	cd23-46	CeDres	18.03.2010	Blood	Not MDR
	cd23-70	CeDres	13.04.2010	Pus	Not MDR
	cd25-14	CeDres	10.10.2010	Urine	Not MDR
	PAC8-8	R-Debré	01.02.2007	CF	
	Ev8	LNSP	05.10.2010	Waste water	ND
	Ln2	LNSP	01.11.2009	Clinical	ND
	cd12-22	CeDres	08.10.2004	Pus	Not MDR
	cd23-45	CeDres	18.03.2010	Pus	Not MDR
	cd24-31	CeDres	14.05.2010	Urine	Not MDR
	cd24-79	CeDres	03.06.2010	Pleural exudate	Not MDR
	cd21-18	CeDres	15.09.2009	Pus	XDR
